# Supplementary material for: SKA2 regulated hyperactive secretory autophagy drives neuroinflammation-induced neurodegeneration
Source: Nat Commun. 2024 Mar 25;15:2635. doi: 10.1038/s41467-024-46953-x (PMC10963788; doi:10.1038/s41467-024-46953-x)
Supplement: Supplementary file 1 — Supplementary Information [file 41467_2024_46953_MOESM1_ESM.pdf]

Supplementary Information

Supplemental Figures and Legends

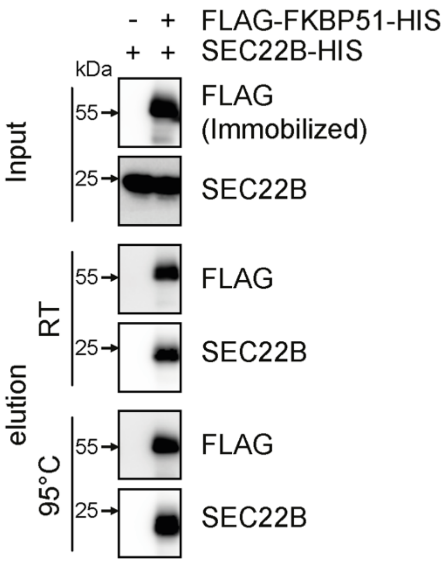

Figure S1. Pull-down assays confirmed a direct protein-protein interaction between FKBP5 and SEC22B. Purified FLAG-FKBP5-HIS was immobilized on anti-FLAG-conjugated magnetic beads and incubated with purified SEC22B-HIS. After washing, bound proteins were eluted first in sodium dodecyl sulfate–polyacrylamide gel electrophoresis (SDS-PAGE) loading buffer at room temperature, and then at 95 °C, also in SDS-PAGE loading buffer (replicated in 3 independent *in vitro* experiments).

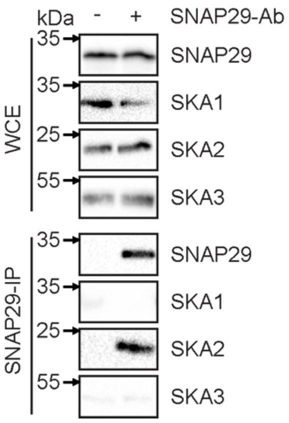

Figure S2. SKA1, SKA2 and SKA3 co-immunoprecipitation (co-IP) experiments (SNAP29-IP) in tissue homogenates from the mouse hippocampus. Co-IP experiments did not reveal any associations of SKA2's partner proteins SKA1 and SKA3 of the SKA complex with SNAP29 (n=3 mice).

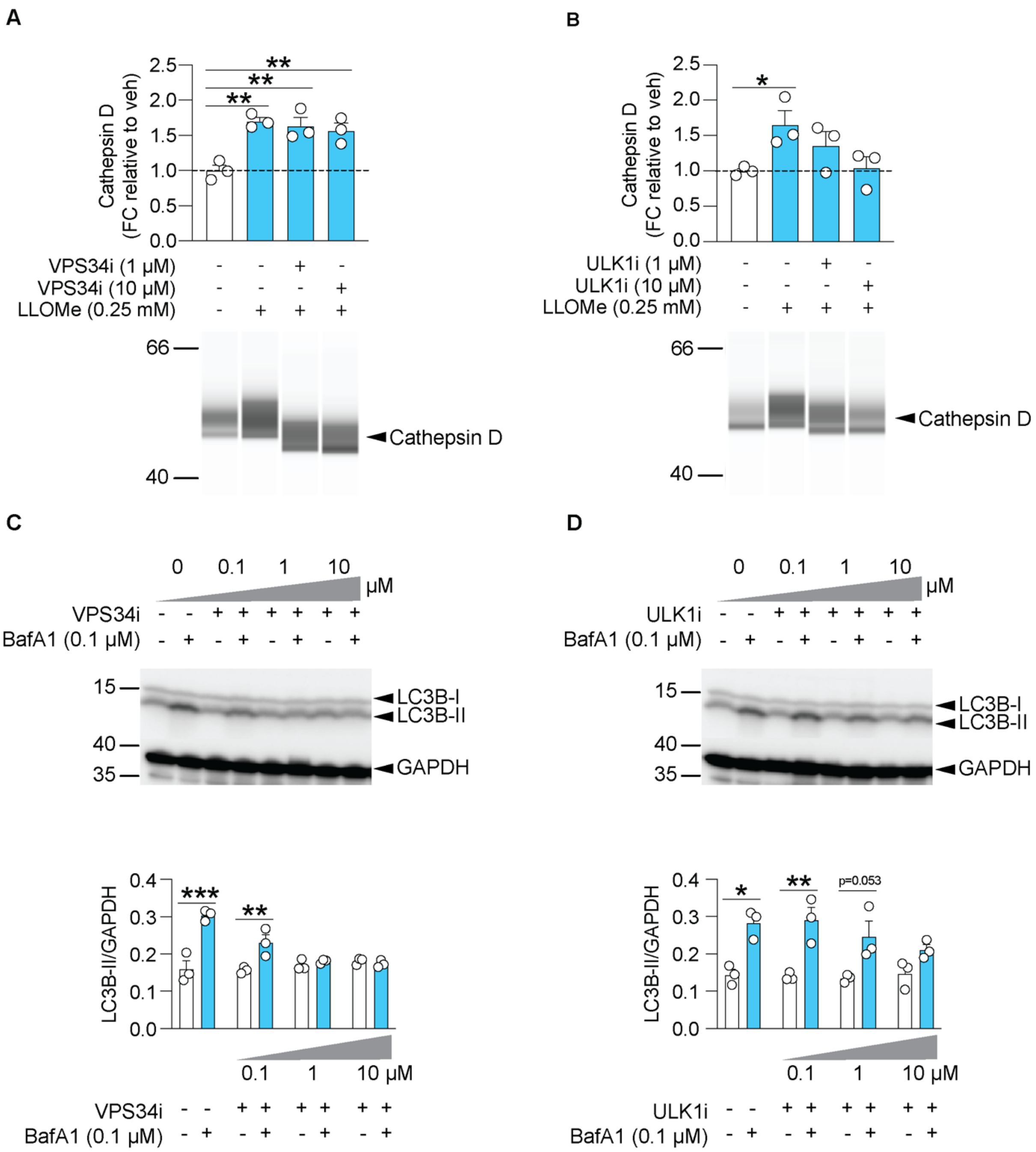

Figure S3. VPS34 inhibitor (VPS34i; SAR405) and ULK1 inhibitor (ULK1i; MRT68921) attenuate LLOMe-induced secretory autophagy and autophagic flux in SIM-A9 cells. (A) Cathepsin D secretion (FC relative to veh) after a 4-hour treatment of SIM-A9 cells with LLOMe (0.25 mM) alone to induce secretory autophagy or with VPS34i at 1 and 10 µM (PIK3C3/ Vps34 inhibition) in combination with 0.25 mM LLOMe (one-way ANOVA, $F_{3, 8} = 11.74$, $p = 0.0027$; Dunnett's multiple comparisons post hoc test: vehicle vs LLOMe, p =

0.002, vehicle vs LLOMe-VPS34i-1μM p = 0.0036, vehicle vs LLOMe-VPS34i-10μM p = 0.0068; n = mean derived from 3 independent *in vitro* experiments). (B) Cathepsin D secretion (FC relative to veh) after a 4-hour treatment of SIM-A9 cells with LLOMe (0.25 mM) alone to induce secretory autophagy or with ULK1i at 1 and 10 μM in combination with 0.25 mM LLOMe (one-way ANOVA, $F_{3, 8} = 3.665$, $p = 0.0630$; Dunnett's multiple comparisons post hoc test: vehicle vs LLOMe, p = 0.0483, vehicle vs LLOMe- ULK1i-1μM p = 0.3198, vehicle vs LLOMe- ULK1i-10μM p = 0.9940; n = mean derived from 3 independent *in vitro* experiments). (C) LC3B-II/ GAPDH levels determined in autophagic flux assays using bafilomycin A1 (BafA1, 0.1 μM) in SIM-A9 cells subjected to increasing concentrations (0.1, 1 and 10 μM) of VPS34i (one-way ANOVA, $F_{7, 16} = 16.69$, $p < 0.0001$; Tukey's multiple comparisons post hoc test: vehicle vs BafA1, p < 0.0001, VPS34i-0.1μM vs VPS34i-0.1μM-BafA1 p = 0.0100; n = mean derived from 3 independent *in vitro* experiments). (D) LC3B-II/ GAPDH levels determined in autophagic flux assays using bafilomycin A1 (0.1 μM) in SIM-A9 cells subjected to increasing concentrations (0.1, 1 and 10 μM) of ULK1i (one-way ANOVA, $F_{7, 16} = 8.408$, $p = 0.0002$; Tukey's multiple comparisons post hoc test: vehicle vs BafA1, p = 0.0160, ULK1i -0.1μM vs ULK1i -0.1μM-BafA1, p = 0.0051, ULK1i -1μM vs ULK1i-1μM-BafA1, p = 0.0530; n = mean derived from 3 independent *in vitro* experiments). Data are presented as mean + SEM. * = p < 0.05; ** = p < 0.01; *** = p < 0.001. Source data are provided as a Source Data file.

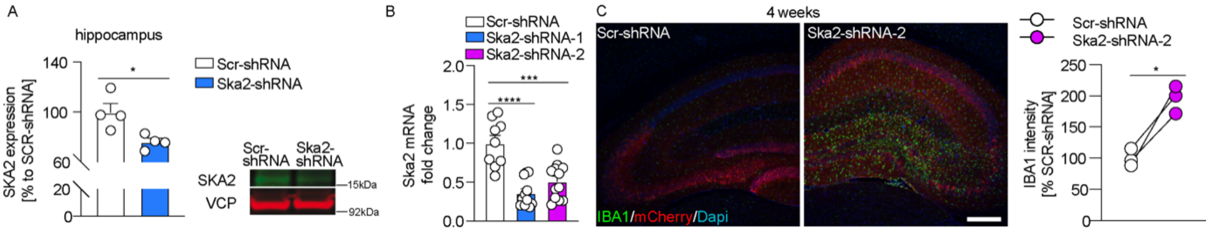

Figure S4. Viral-mediated knockdown of *Ska2* (Ska2-shRNA-1-AAV) in mouse the hippocampus. (A) Viral-mediated knockdown of *Ska2* (Ska2-shRNA-1-AAV) in the hippocampus leads to significantly decreased SKA2 expression (unpaired, two tailed t-test: $t_6 = 3.13$, $p = 0.0230$; n = 4 mice per group). (B) Ska2 mRNA expression is significantly decreased following transfection with Ska2-shRNA-1 or Ska2-shRNA-2 in Neuro2a cells (one-way analysis of variance (ANOVA), $F_{2, 29} = 19.60$, $p < 0.0001$; Tukey's multiple comparisons post hoc test: scr-shRNA vs Ska2-shRNA-1, $p < 0.0001$, scr-shRNA vs Ska2-shRNA-2, $p = 0.0002$; scr-shRNA: n = 9, Ska2-shRNA-1: n = 11, Ska2-shRNA-2: n = 12). (C) Viral-mediated knockdown of *Ska2* (Ska2-shRNA-2-AAV) leads to increased IBA1 expression 4 weeks after viral injection (paired t-test: $t_2 = 5.981$, $p = 0.0268$; n = 3 mice. Data are presented as mean + SEM. * = $p < 0.05$; *** = $p < 0.001$; **** = $p < 0.0001$. Scale bar represents 250 µm. Source data are provided as a Source Data file.

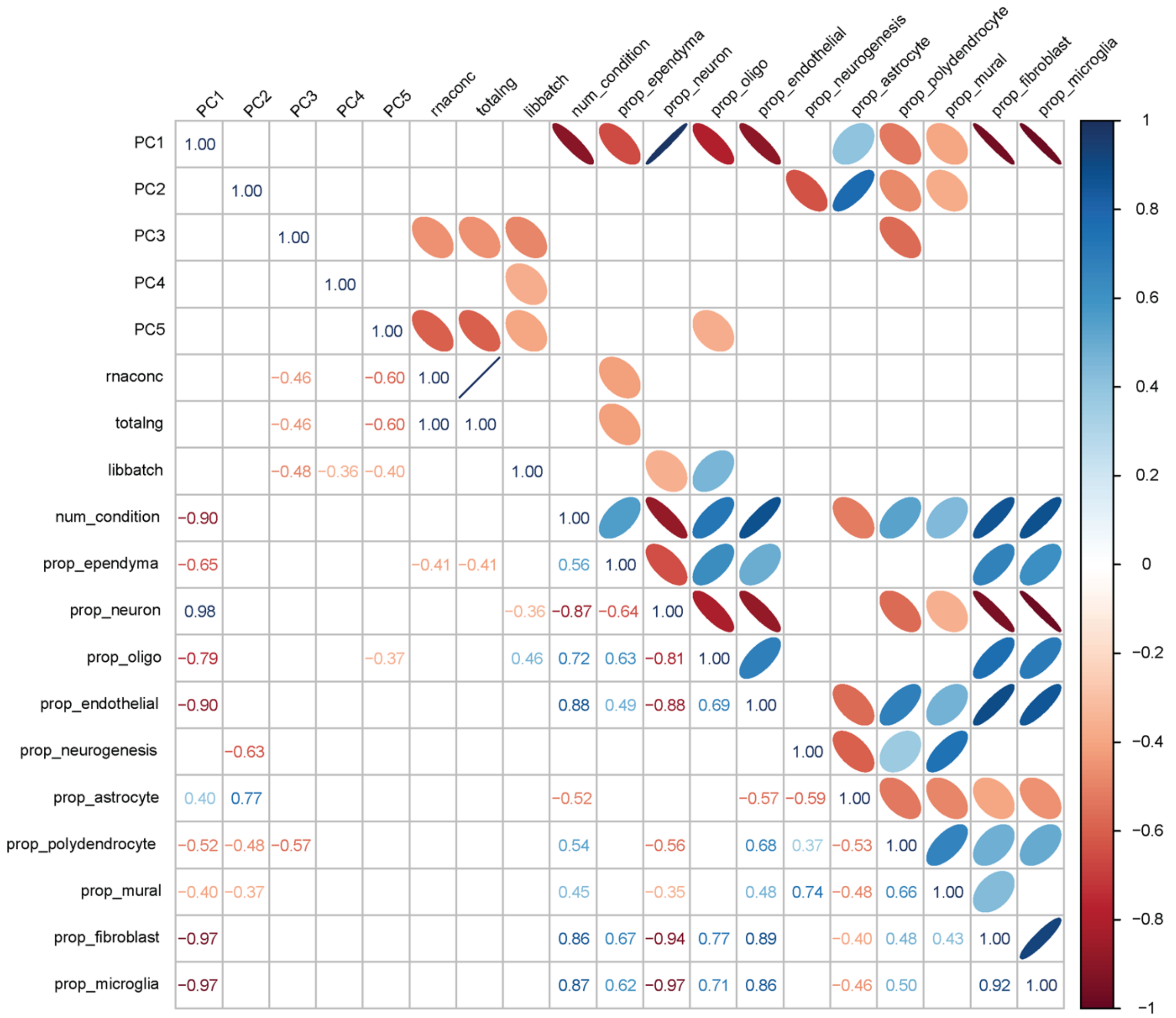

Figure S5. Correlation analysis of technical and biological variables with principal components. Depicted are technical and biological variables in the data, with the color, shape, and direction of the ovals in the top right side and numerical values on the bottom left side indicating the correlation value. The top 5 principal components explain >90% of the variance in the gene expression data. The total RNA concentration denoted by rnaconc and totalng is the amount of RNA that is used as input for RNAseq. Libbatch represents the library batches. Num_condition denotes the different conditions of the Ska2 knockdown at 2 and 4 weeks, including the controls. Prop_[celltypes] are the proportions of cell types obtained from the deconvolution analysis. PC1 is highly correlated with the groups and proportion cell types; this indicated the strong effect of Ska2 knockdown. Additionally, libbatch is correlated with PC3 until PC5 and therefore added as a covariate in the differential expression analysis. The

proportion microglia is strongly correlated with other cell types and is therefore representative for cell type composition.

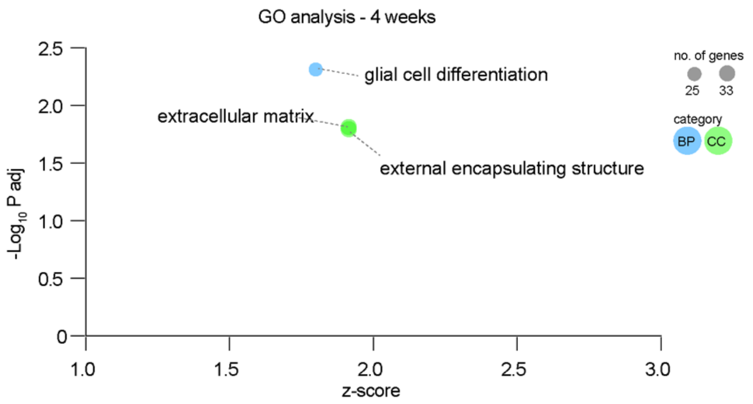

Figure S6. Gene ontology (GO) enrichment analysis with differentially expressed genes at 4 weeks. Enriched GO terms. Circle size is proportional to the number of genes. BP, biological process, CC, cellular component. Source data are provided as a Source Data file.

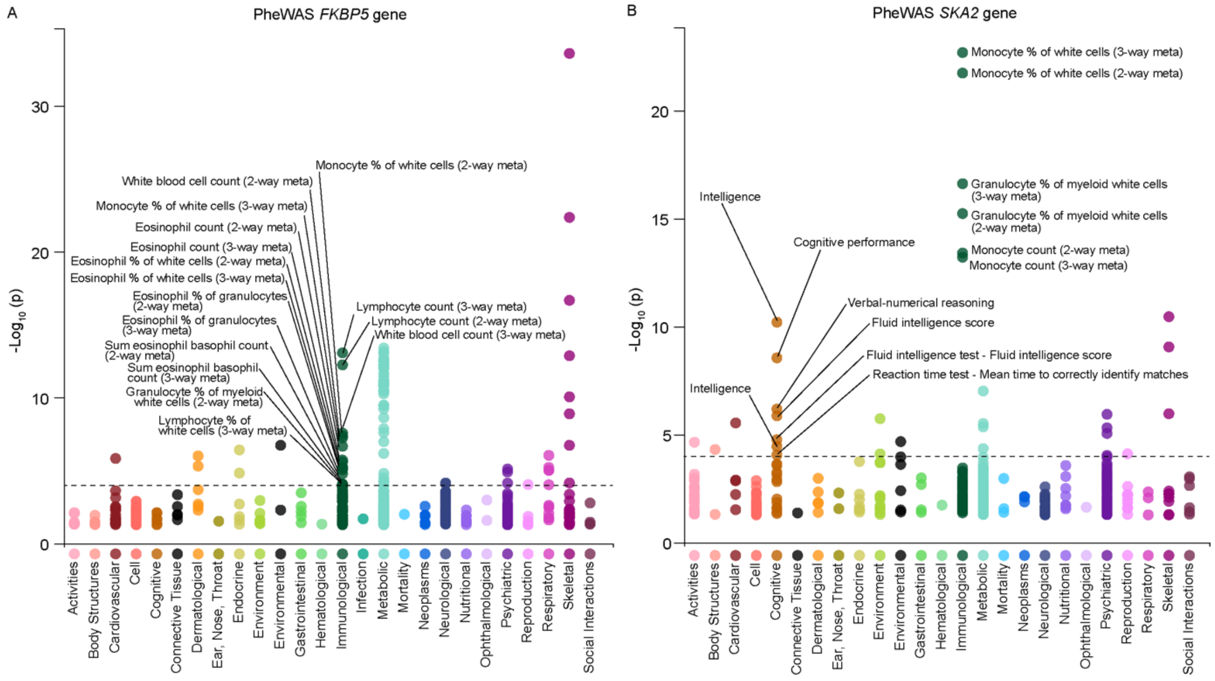

Figure S7. PheWAS plots of phenotypes associated with the *FKBP5* (A) and *SKA2* (B) gene. The x axes represent phenotypes, and the y axes represent the -$\log_{10}$ of uncorrected p values. The dashed lines indicate the experiment-wide threshold to survive Bonferroni correction (*FKBP5*: $p_{\text{-Log10}} < 4.016$ and *SKA2*: $p_{\text{-Log10}} < 3.876$). Each dot represents one phenotype, and the colors indicate their according traits. Representative top findings are annotated in the figure.

Full-lane blots.

S1

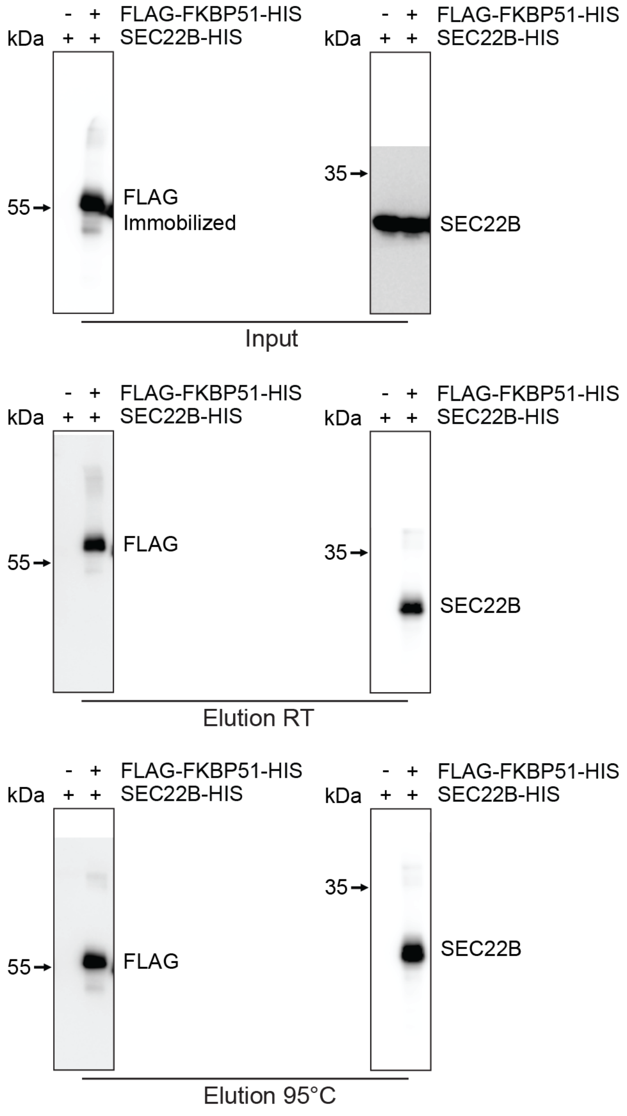

S2

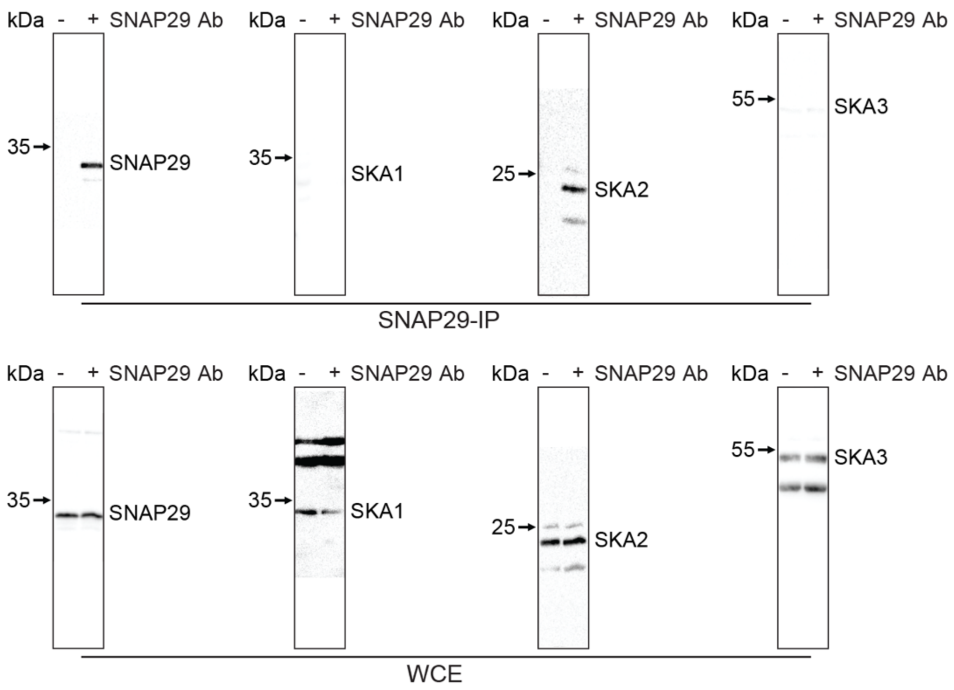
kDa - + SNAP29 Ab
35
SNAP29
kDa - + SNAP29 Ab
35
SKA1
kDa - + SNAP29 Ab
25
SKA2
kDa - + SNAP29 Ab
55
SKA3
SNAP29-IP
kDa - + SNAP29 Ab
35
SNAP29
kDa - + SNAP29 Ab
35
SKA1
kDa - + SNAP29 Ab
25
SKA2
kDa - + SNAP29 Ab
55
SKA3
WCE

89

S3C

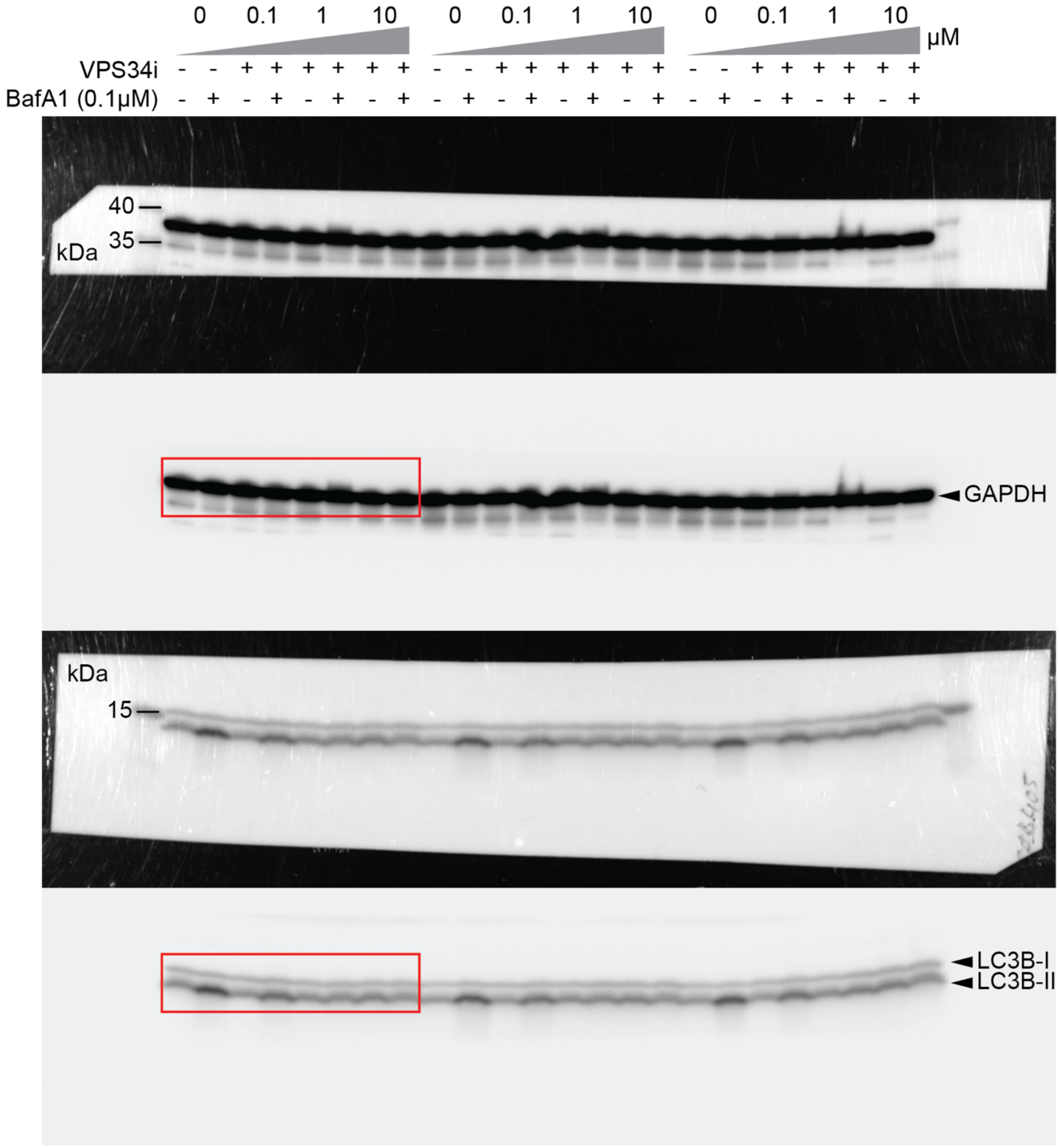
0 0.1 1 10 0 0.1 1 10 0 0.1 1 10
μM
VPS34i
BafA1 (0.1μM)
40
35
kDa
GAPDH
kDa
15
LC3B-I
LC3B-II

S3D

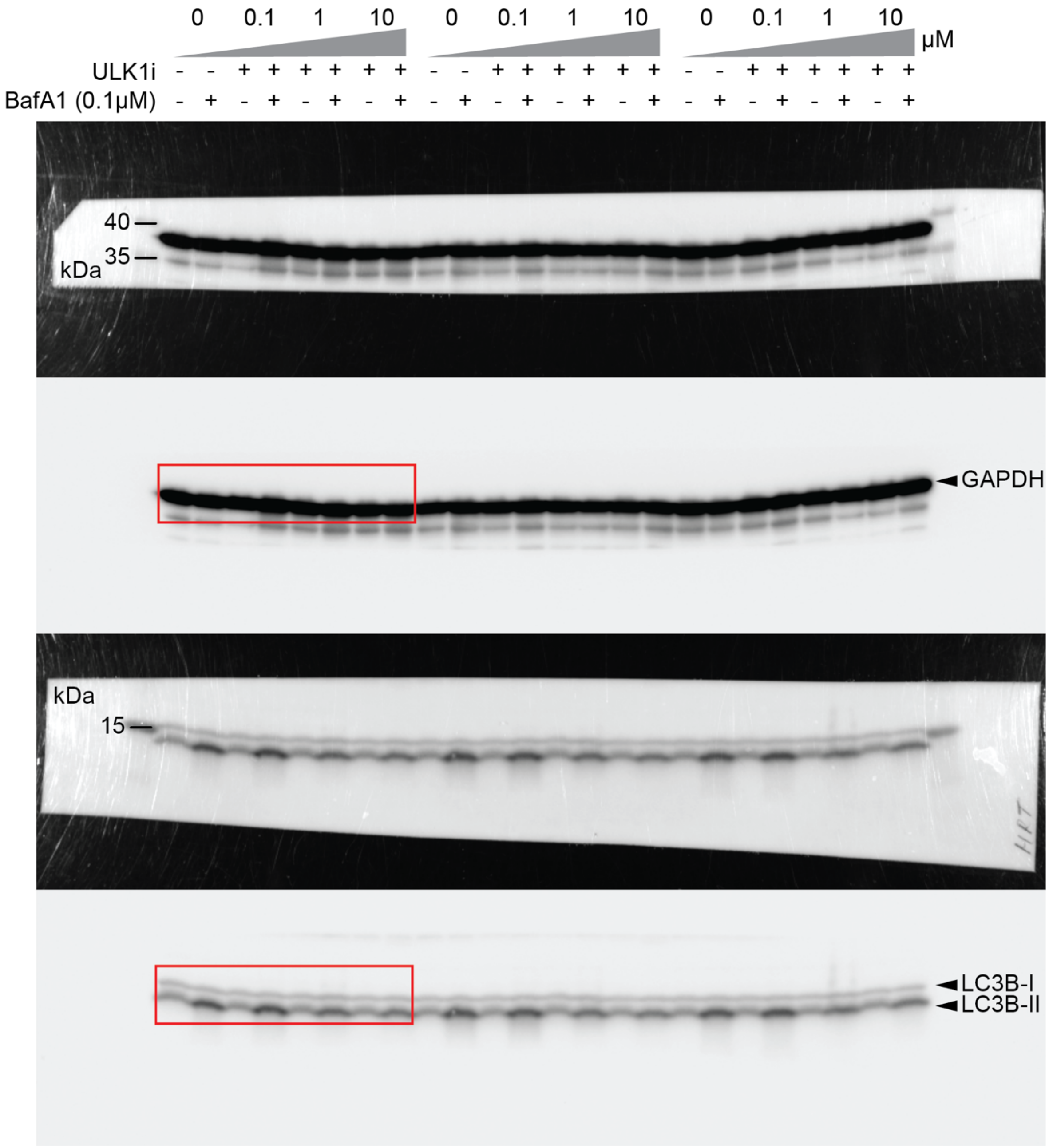
0 0.1 1 10 0 0.1 1 10 0 0.1 1 10
μM
ULK1i - - + + + + + + - - + + + + + + - - + + + + + +
BafA1 (0.1μM) - + - + - + - + - + - + - + - + - + - + - + - +
40
35
kDa
GAPDH
kDa
15
LC3B-I
LC3B-II

S4A

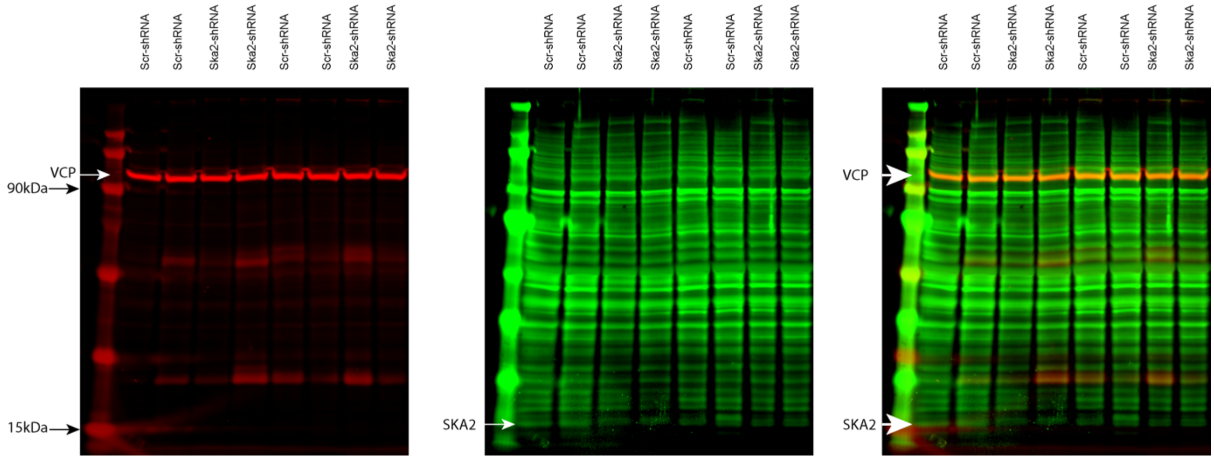
Scr-shRNA
Scr-shRNA
Ska2-shRNA
Ska2-shRNA
Scr-shRNA
Scr-shRNA
Ska2-shRNA
Ska2-shRNA
VCP
90kDa
15kDa
SKA2
VCP
SKA2
