# Supplementary material for: SKA2 regulated hyperactive secretory autophagy drives neuroinflammation-induced neurodegeneration
Source: Nat Commun. 2024 Mar 25;15:2635. doi: 10.1038/s41467-024-46953-x (PMC10963788; doi:10.1038/s41467-024-46953-x)
Supplement: Supplementary file 3 — Description of Additional Supplementary Files [file 41467_2024_46953_MOESM3_ESM.pdf]

Description of Additional Supplementary Files

File Name: Supplementary Data 1
Description: Differentially expressed genes 2 weeks after viral-mediated knockdown of Ska2.

File Name: Supplementary Data 2
Description: Differentially expressed genes 4 weeks after viral-mediated knockdown of Ska2.

File Name: Supplementary Data 3
Description: Gene ontology enrichment analysis 2 weeks after viral-mediated knockdown of Ska2.

File Name: Supplementary Data 4
Description: Gene ontology enrichment analysis 4 weeks after viral-mediated knockdown of Ska2.

File Name: Supplementary Data 5
Description: Kyoto Encyclopedia of Genes and Genomes pathway enrichment analysis 2 weeks after viral-mediated knockdown of Ska2.

File Name: Supplementary Data 6
Description: Phenome-Wide Association Studies (PheWAS) table of the FKBP5 locus.

File Name: Supplementary Data 7
Description: Phenome-Wide Association Studies (PheWAS) table of the SKA2 locus.

File Name: Supplementary Data 8
Description: Details of human postmortem subjects (Immunoprecipitation).

File Name: Supplementary Data 9
Description: Details of human postmortem subjects (Immunohistochemistry).

File Name: Supplementary Data 10
Description: Details of human postmortem subjects (Alzheimer’s disease discovery cohort (HIP)).

File Name: Supplementary Data 11

Description: Details of human postmortem subjects (Alzheimer's disease replication cohort (PFC)).
