# Supplementary material for: SKA2 regulated hyperactive secretory autophagy drives neuroinflammation-induced neurodegeneration
Source: Nat Commun. 2024 Mar 25;15:2635. doi: 10.1038/s41467-024-46953-x (PMC10963788; doi:10.1038/s41467-024-46953-x)
Supplement: Supplementary file 5 — Reporting Summary [file 41467_2024_46953_MOESM5_ESM.pdf]

nature portfolio

Corresponding author(s): Hartmann, Ressler, Gassen
Last updated by author(s): Feb 27, 2024

Reporting Summary

Nature Portfolio wishes to improve the reproducibility of the work that we publish. This form provides structure for consistency and transparency in reporting. For further information on Nature Portfolio policies, see our Editorial Policies and the Editorial Policy Checklist.

Statistics

For all statistical analyses, confirm that the following items are present in the figure legend, table legend, main text, or Methods section.

n/a	Confirmed	
☐	☒	The exact sample size (*n*) for each experimental group/condition, given as a discrete number and unit of measurement
☐	☒	A statement on whether measurements were taken from distinct samples or whether the same sample was measured repeatedly
☐	☒	The statistical test(s) used AND whether they are one- or two-sided<br>*Only common tests should be described solely by name; describe more complex techniques in the Methods section.*
☐	☒	A description of all covariates tested
☐	☒	A description of any assumptions or corrections, such as tests of normality and adjustment for multiple comparisons
☐	☒	A full description of the statistical parameters including central tendency (e.g. means) or other basic estimates (e.g. regression coefficient) AND variation (e.g. standard deviation) or associated estimates of uncertainty (e.g. confidence intervals)
☐	☒	For null hypothesis testing, the test statistic (e.g. *F*, *t*, *r*) with confidence intervals, effect sizes, degrees of freedom and *P* value noted<br>*Give P values as exact values whenever suitable.*
☒	☐	For Bayesian analysis, information on the choice of priors and Markov chain Monte Carlo settings
☐	☒	For hierarchical and complex designs, identification of the appropriate level for tests and full reporting of outcomes
☐	☒	Estimates of effect sizes (e.g. Cohen's *d*, Pearson's *r*), indicating how they were calculated

Our web collection on statistics for biologists contains articles on many of the points above.

Software and code

Policy information about availability of computer code

Data collection	Behavioral experiments were recorded and analyzed using the automated video-tracking system ANYmaze (6.33). An Olympus BX61 interfaced with StereoInvestigator v.2019 (MBF Biosciences, Willinston, VT) and a Zeiss LSM 880 confocal microscope interfaced with Zen imaging software (ZEN 2.3 SP1 and ZEN 2.6) were used to acquire and analyze human postmortem IHC images. A Leica SP8 confocal microscope interfaced with a Leica Application Suite X (LAS X) software was used to acquire mouse IHC images. Images were quantified using ImageJ (v.1.52p). Images for assessment of ASC specks in PFA-fixed SIM-A9 cells were acquired using the VisiScope CSU-W1 spinning disk confocal microscope and the VisiView Software (Visitron Systems GmbH). Immunoprecipitates of protein extracts obtained from human post mortem brains were analyzed using capillary-based immunoassays (Jess, ProteinSimple). Microdialysates obtained from in vivo acute stress experiments were analyzed using capillary-based immunoassays (Jess, ProteinSimple). Compass software was used for quantification of obtained signals (ProteinSimple, Bio-Techne).
Data analysis	Software used for data analysis included: GraphPad Prism (v.10.0), JMP Pro (v.14SW), R (v.4.1.2), DESeq2 (v.1.34.0), bcbio-nextgen (v.1.2.0), MultiQC (v.1.8), clusterProfiler (v.4.2.2), org.Mm.eg.db (v.4.0), GOplot (v.1.0.2), msigdbr (v.7.5.1), MuSiC (v.1.0.0), StereoInvestigator (v.2019), ImageJ (v.1.52p), ANYmaze (6.33), Zen imaging software (ZEN 2.3 SP1 and ZEN 2.6), Leica Application Suite X (LAS X) software, VisiView Software (Visitron Systems GmbH), Compass software (ProteinSimple, Bio-Techne).

For manuscripts utilizing custom algorithms or software that are central to the research but not yet described in published literature, software must be made available to editors and reviewers. We strongly encourage code deposition in a community repository (e.g. GitHub). See the Nature Portfolio guidelines for submitting code & software for further information.

Data

Policy information about availability of data

All manuscripts must include a data availability statement. This statement should provide the following information, where applicable:

- Accession codes, unique identifiers, or web links for publicly available datasets
- A description of any restrictions on data availability
- For clinical datasets or third party data, please ensure that the statement adheres to our policy

The M. musculus genome (GRC38/mm10) used to map the raw reads of the RNA sequencing data is available at: https://www.ncbi.nlm.nih.gov/datasets/genome/GCF_000001635.20/
The RNA sequencing data following Ska2 knockdown (Figure 6, Fig. S5 and Fig. S6) generated in this study are available at Gene Expression Omnibus (GEO): GSE181203.
https://www.ncbi.nlm.nih.gov/geo/query/acc.cgi?acc=GSE181203

Single-cell RNA sequencing data used for the deconvolution analysis are available at GEO: GSE116470.
https://www.ncbi.nlm.nih.gov/geo/query/acc.cgi?acc=GSE116470

Original data for Figure S7A and B (Phenotypic data for the FKBP5 and SKA2) genes were publicly available from the Atlas of GWAS Summary Statistics and can be downloaded at http://atlas.ctglab.nl/PheWAS.

Source data are provided with this paper.

Research involving human participants, their data, or biological material

Policy information about studies with human participants or human data. See also policy information about sex, gender (identity/presentation), and sexual orientation and race, ethnicity and racism.

Reporting on sex and gender	Biological sex was determined through clinical records. Sex-specific analyses were not performed, due to the low sample size but sex was included as a covariate in all statistical models.
Reporting on race, ethnicity, or other socially relevant groupings	We did not use any socially relevant variables (e.g. race, ethnicity etc.) in our studies.
Population characteristics	Alzheimer's disease discovery cohort: Tissue blocks containing the hippocampus from donors with Alzheimer's disease (n = 7; 4M/3F) and healthy control subjects (n = 13; 8M/5F) were used for western blotting and co-immunoprecipitation. All subjects were characterized clinically and neuropathologically as above. 'Control' cases had Braak & Braak scores of 0-II, sparse plaque pathology, and were rated as having low probability of AD. AD cases had Braak & Braak scores of III-VI and were rated as having intermediate or high probability of AD. Neither group presented with additional relevant neuropathological findings. Groups were matched based on demographic factors age (Ctrl:*76.5±2.2, AD: *77.9±0.6), sex (Ctrl: 8M/5F, AD: 4M/3F), postmortem interval (PMI) (Ctrl: *21.4±0.8, AD: *17.7±2.0), Braak & Braak score (Ctrl: 0-II, AD: III-VI), hemisphere (Ctrl: 8R/5L, AD: 4R/3L), brain weight (Ctrl: *1268.1±50.2, AD: *1182.9±58). Alzheimer's disease replication cohort: Fresh, frozen tissue was taken from the superior frontal gyrus (Brodmann area 8) of the frontal cortex from 77 brains (AD: n = 40 (14M/26F), Ctrl: n = 37 (10M/27F)) of donors who were participants of a large prospective cognitive aging cohort known as the University of Manchester Longitudinal Study of Cognition in Normal Healthy Old Age Cohort (UMLCHA). Samples were used for western blotting. AD neuropathology was determined as described above. Groups were matched based on demographic factors age (Ctrl: *88.9±1.0, AD: *88.2±1.0), sex (Ctrl: 10M/27F, AD: 14M/26F), postmortem interval (PMI) (Ctrl: *75.8±8.0, AD: *84.0±7.1), Braak & Braak score (Ctrl: 0-II, AD: III-VI), hemisphere (Ctrl: 37R, AD: 40R), brain weight (Ctrl: *1177.0±28.8, AD: *1232.0±25.9).
Recruitment	Alzheimer's disease discovery cohort: The HBTRC recruits brain donors across the U.S.A. using a community-based approach. Prospective donors may register during life and/or are referred by clinicians and social workers. Donors with a broad range of brain disorders, as well as unaffected by brain disorders, are accepted regardless of their gender, age, sex, race, class, religion, ethnicity, language, sexual orientation, or gender identity. However, the donor demographics reflect a largely Caucasian population and average donor age approximately around 60 - 65 years of age. Exclusionary criteria are stroke and penetrating injuries to the head, serum positive for HIV, Hep B and C, prion disorders and extended time on respirator. Alzheimer's disease replication cohort: Brain donors were participants of a large prospective cognitive aging cohort known as the University of Manchester Longitudinal Study of Cognition in Normal Healthy Old Age Cohort (UMLCHA) (Rabbitt et al. 2004; Robinson et al . 2018).<br>Rabbitt, P. M. A. et al. The University of Manchester Longitudinal Study of Cognition in Normal Healthy Old Age, 1983 through 2003. Aging, Neuropsychol. Cogn. 11, 245-279 (2004).<br>Robinson, A. C., Davidson, Y. S., Horan, M. A., Pendleton, N. & Mann, D. M. A. Pathological Correlates of Cognitive Impairment in The University of Manchester Longitudinal Study of Cognition in Normal Healthy Old Age. J. Alzheimers. Dis. 64, 483-496 (2018).
Ethics oversight	According to NIH guidelines, research using de-identified postmortem tissue is not considered as human subject research. Approval for donation and use of the biological samples for postmortem analyses has been obtained from the Harvard Brain Tissue Resource Center / NIH NeuroBioBank (HBTRC/NBB) through the Mass General Brigham IRB under protocol

#2015P002028, and from the Manchester Brain Bank through the Newcastle & North Tyneside 1 Research Ethics Committee (19/NE/0242).

Note that full information on the approval of the study protocol must also be provided in the manuscript.

Field-specific reporting

Please select the one below that is the best fit for your research. If you are not sure, read the appropriate sections before making your selection.

- [x] Life sciences
- [ ] Behavioural & social sciences
- [ ] Ecological, evolutionary & environmental sciences

For a reference copy of the document with all sections, see nature.com/documents/nr-reporting-summary-flat.pdf

Life sciences study design

All studies must disclose on these points even when the disclosure is negative.

Sample size	Experiments were informed by prior research indicating n = 3-7 biological replicates were sufficient for detecting significant differences for effect size estimations. For behavioral experiments, animal numbers were based on historical data, using G*Power 3 for estimation.
Data exclusions	In the behavioral analysis of the novel object recognition test, one mouse of the SKA2-KD group (Ska2-shRNA) was excluded based on Grubbs' outlier test. For the EPM behavioral data, one mouse of the ctrl group (Scr-shRNA) fell off the open arm during testing and was excluded from the analysis. No other data were excluded.
Replication	All experiments were performed in at least three biological replicates. All attempts at replication were successful.
Randomization	Experimental groups were assigned in a semi-randomized manner to ensure a balanced distribution across treatments. This initial randomization was deemed sufficient, as parallel processing of samples minimized potential variability. For AD and control human postmortem samples, selection was based on matching co-variates (age, sex, postmortem interval (PMI), brain hemisphere, and brain weight) and Braak & Braak staging for disease progression.
Blinding	All experiments and data analyses were completed by an experimenter blinded to the group allocation during data collection.

Reporting for specific materials, systems and methods

We require information from authors about some types of materials, experimental systems and methods used in many studies. Here, indicate whether each material, system or method listed is relevant to your study. If you are not sure if a list item applies to your research, read the appropriate section before selecting a response.

Materials & experimental systems

n/a	Involved in the study	
[ ]	[x]	Antibodies
[ ]	[x]	Eukaryotic cell lines
[x]	[ ]	Palaeontology and archaeology
[ ]	[x]	Animals and other organisms
[x]	[ ]	Clinical data
[x]	[ ]	Dual use research of concern
[x]	[ ]	Plants

Methods

n/a	Involved in the study	
[x]	[ ]	ChIP-seq
[x]	[ ]	Flow cytometry
[x]	[ ]	MRI-based neuroimaging

Antibodies

Antibodies used	Primary antibodies used:<br>LC3B (1:1,000, 2775, Cell Signaling Technology)<br>GAPDH (1:1,000, 5174, Cell Signaling Technology)<br>Cathepsin D AF4G5 (LF-MA0321, 1:50, Abfrontier)<br>IL1B (1:50, Gene Tex, GTX74034)<br>FKBP5 (1:1000, Bethyl, A301-430A)<br>FKBP5 (1:1000, Cell Signaling Technology, #12210)<br>ACTIN (1:5000, Santa Cruz Biotechnology, sc-1616)<br>GAPDH (1:8000, Millipore CB1001)<br>SNAP29 (1:1000, Sigma, SAB1408650)<br>SNAP23 (1:1000, Sigma, SAB2102251)<br>STX3 (1:1000, Sigma, SAB2701366)<br>STX4 (1:1000, Cell Signaling Technology, 67657)<br>SEC22B (1:1000, Abcam, ab181076)

SKA2 (1:1000, Thermo Fisher, PAS-20818)
SKA2 (1:300/1:500, Millipore-Sigma, SAB3500102)
SKA1 (1:1000, Thermo Fisher, PA5-20817)
SKA3 (1:1000, Thermo Fisher, PA5-20819)
GSDMD (1:1000, Cell Signaling Technology, 39754)
VCP (1:10000, Abcam, Ab11433)
NEK7 (1:50, Abcam, Ab133514)
NLRP3 (1:50, Cell Signaling Technology, 15101)
HIS (1:5000, Cell Signaling Technology, #12698)
FLAG M2 (1:10000, Sigma, A8592)
FLAG M2 (Sigma, F3165; used for pull-down assay)
NeuN (1:1000, Synaptic Systems, 266004)
IBA1 (1:1000, FUJIFILM Cellular Dynamics, 019-19471)
GFAP (1:1000, Cell Signaling Technology, 12389)
ASC (1:200, AdipoGen, AG-25B-0006-C100)
CASP-1 (1:1000, Santa Cruz, sc-56036)
anti-mCherry (1:1000, Millipore Sigma, AB356481)
IBA1 (1:500, FUJIFILM Cellular Dynamics 013-27593)
CamKII (1:500, Abcam, ab22609)
SNAP29 (1:100, Santa Cruz Biotechnology, sc-390602)

Secondary antibodies used:
horseradish peroxidase-conjugated secondary antibody (1:10,000, 7074, Cell Signaling Technology)
horseradish peroxidase-conjugated secondary antibody at 1:100 (7076, Cell Signaling Technology)
anti-rabbit-IgG (1:1000, Cell Signaling, 7074)
antimouse-IgG (1:1000, Cell Signaling, 7076)
IRDyes 800CW donkey anti-Rabbit (1:20,000, LI-COR Biosciences, 926-32213)
IRDye 680RD goat-anti-mouse (1:20,000, LI-COR Biosciences, 926-68070)
Alexa Fluor goat anti-mouse 647 (1:300; Invitrogen, A-21235)
Alexa Fluor donkey anti-rabbit 488 (1:300/1:1000; Invitrogen A-21206)
Alexa Fluor goat anti-chicken 594 (1:1000; Invitrogen, A-11042),
Alexa Fluor goat anti-Guinea Pig 594 (1:1000, Invitrogen, A-11076)
Alexa Fluor goat anti-rabbit 555 (1:300, Invitrogen, A-32732)

Validation

The antibodies were validated by the manufacturer.

LC3B 2775 (validated for WB; species reactivity H M R)
GAPDH 5174 (validated for WB IHC IF; species reactivity H M R Mk)
Cathepsin D AF4G5 LF-MA0321 (validated for WB; species reactivity H M R)
IL1B GTX74034 (validated for WB ICC/IF IHC-P IHC-Fr ELISA IHC; species reactivity H M R)
FKBP5 A301-430A (validated for WB, IP, IF; species reactivity H, M)
FKBP5 #12210 (validated for WB, IP; species reactivity H, M, R, Mk)
ACTIN sc-1616 (validated for WB; species reactivity H M R)
GAPDH CB1001 (validated for WB IF ELISA; species reactivity H M R Mk Canine Chicken, Fish Porcine Frog Rb)
SNAP29 SAB1408650 (validated for WB; species reactivity for H R)
SNAP23 SAB2102251 (validated for WB; species reactivity H)
STX3 SAB2701366 (validated for WB IF; species reactivity H M R)
STX4 67657 (validated for WB, IP, IF; species reactivity H)
SEC22B ab181076 (validated for WB IP IF; species reactivity H M R)
SKA2 PAS-20818 (validated for WB; species reactivity H M)
SKA2 SAB3500102 (validated for WB ELISA; species reactivity H M)
SKA1 PA5-20817 (validated for WB; species reactivity H M R)
SKA3 PA5-20819 (validated for WB; species reactivity H M R),
GSDMD 39754 (validated for WB IHC; species reactivity H M R)
VCP Ab11433 (validated for WB IHC ELISA; species reactivity H M R)
NEK7 Ab133514 (validated for WB IP; species reactivity H M R)
NLRP3 15101 (validated for WB IP; species reactivity H M)
HIS #12698 (validated for WB, IP; species reactivity all)
FLAG M2 A8592 (validated for WB, IHC; species reactivity all)
FLAG M2 (F3165 validated for WB; species reactivity all).
NeuN 266004 (validated for IHC; species reactivity H M R)
IBA1 019-19471 (validated for IHC ICC; species reactivity H M R)
GFAP 12389 (validated with WB IHC; species reactivity H M R)
ASC (AG-25B-0006-C100 (validated for IHC WB IP; species reactivity H M)
CASP-1 sc-56036 (validated for WB IP IHC; species reactivity H M R)
anti-mCherry AB356481 (validated for IHC WB)
IBA1 013-27593 (validated for IHC; species reactivity H)
CamKII ab22609 (validated for IHC WB; species reactivity H M R)

	SNAP29 sc-390602 (validated for WB; species reactivity H)

Eukaryotic cell lines

Policy information about cell lines and Sex and Gender in Research

Cell line source(s)	N2a cells (ATCC, CCL-131)<br>SIM-A9 wild type cells (Kerafast, END001)<br>SIM-A9 Fkbp5 knockout cells, SIM-A9 Sec22b knockout cells (Martinelli et al. Nat. Comm, 2021)
Authentication	Commercial cell lines were not further authenticated in the lab.
Mycoplasma contamination	All cell lines tested negative for mycoplasma contamination
Commonly misidentified lines (See ICLAC register)	No commonly misidentified cell lines were used in the study

Animals and other research organisms

Policy information about studies involving animals; ARRIVE guidelines recommended for reporting animal research, and Sex and Gender in Research

Laboratory animals	Male mice, aged 2 to 4 months, were used for all experiments. For experiments in wild type animals, C57BL/6J mice were obtained from The Jackson Laboratory (Bar Harbor, ME, USA). For in vivo brain microdialysis experiments, C57BL/6NCrl mice (Martinsried, Germany) as well as global Fkbp5-/- mice (Hartmann et al. 2012 Neuropharmacology) and respective wild type controls were used. Neonatal (P7-9) Thy1-GFP M mice (sex not determined) with a sparse expression of green fluorescent protein (GFP) in principal neurons in cortex and hippocampus, Jackson Laboratory Stock #007788) were used in organotypic hippocampal slice cultures (OHSC) experiments.
Wild animals	This study did not involve wild animals.
Reporting on sex	Sex was not a factor considered in the animal study design, and as a result, sex-based analyses were not performed since only male mice were used in the experiments. The sex of the animals is explicitly stated in both the abstract and the methods section of the manuscript.
Field-collected samples	This study did not involve field-collected samples.
Ethics oversight	All experiments conformed to National Institutes of Health guidelines and were carried out in accordance with the European Communities' Council Directive 2010/63/EU and the McLean Hospital Institutional Animal Care and Use Committee. All efforts were made to minimize animal suffering during the experiments. The protocols were approved by the committee for the Care and Use of Laboratory animals of the Government of Upper Bavaria, Germany or by the local Institutional Animal Care and Use Committee, respectively.

Note that full information on the approval of the study protocol must also be provided in the manuscript.

Plants

Seed stocks	Not applicable.
Novel plant genotypes	Not applicable.
Authentication	Not applicable.
